# Voltage modulates the effect of μ‐receptor activation in a ligand‐dependent manner

**DOI:** 10.1111/bph.15070

**Published:** 2020-05-19

**Authors:** Julia G. Ruland, Sina B. Kirchhofer, Sebastian Klindert, Chris P. Bailey, Moritz Bünemann

**Affiliations:** ^1^ Department of Pharmacology and Clinical Pharmacy Philipps‐University Marburg Germany; ^2^ Department of Pharmacy and Pharmacology University of Bath Bath UK

## Abstract

**Background and Purpose:**

Various GPCRs have been described as being modulated in a voltage‐dependent manner. Opioid analgesics act via activation of μ receptors in various neurons. As neurons are exposed to large changes in membrane potential, we were interested in studying the effects of depolarization on μ receptor signalling.

**Experimental Approach:**

We investigated potential voltage sensitivity of μ receptors in heterologous expression systems (HEK293T cells) using electrophysiology in combination with Förster resonance energy transfer‐based assays. Depolarization‐induced changes in signalling were also tested in physiological rat tissue containing locus coeruleus neurons. We applied depolarization steps across the physiological range of membrane potentials.

**Key Results:**

Studying μ receptor function and signalling in cells, we discovered that morphine‐induced signalling was strongly dependent on the membrane potential (V_M_). This became apparent at the level of G‐protein activation, G‐protein coupled inwardly rectifying potassium channel (K_ir_3.X) currents and binding of GPCR kinases and arrestin3 to μ receptors by a robust increase in signalling upon membrane depolarization. The pronounced voltage sensitivity of morphine‐induced μ receptor activation was also observed at the level of K_ir_3.X currents in rat locus coeruleus neurons. The efficacy of peptide ligands to activate μ receptors was not (Met‐enkephalin) or only moderately ([D‐Ala^2^, N‐Me‐Phe^4^, Gly^5^‐ol]‐enkephalin) enhanced upon depolarization. In contrast, depolarization reduced the ability of the analgesic fentanyl to activate μ receptors.

**Conclusion and Implications:**

Our results indicate a strong ligand‐dependent modulation of μ receptor activity by the membrane potential, suggesting preferential activity of morphine in neurons with high neuronal activity.

**Abbreviations**
aCSF: artificial CSF
DAMGO: [D‐Ala^2^, N‐Me‐Phe^4^, Gly^5^‐ol]‐enkephalin
FRET: Förster Resonance Energy Transfer
GRK: GPCR kinase
HEK293T: HEK cells 293
K_ir_3.X: G‐protein coupled inwardly rectifying potassium channel
LC: locus coeruleus
ME: Met‐enkephalin
V_M_: membrane potential

**What is already known**

- Opioid analgesics differ in potency and side effects and act primarily via μ receptors.

**What this study adds**

- The efficacy of μ receptor activation via opioid analgesics is strongly voltage‐dependent.
- Membrane voltage differentially affects μ receptor activation by morphine, Met‐enkephalin or fentanyl.

**What is the clinical significance**

- The differential voltage‐dependence of opioid analgesics suggests differential drug responses dependent on neuronal activity.

## 1 INTRODUCTION

The opioid μ receptor is a class A GPCR that activates G_i/o_ proteins. Agonists acting at this receptor are not only used to treat moderate to severe pain (Corbett, Henderson, McKnight, & Paterson, 2006) but also abused for their euphorigenic effects (Morgan & Christie, 2011). Due to its powerful analgesic action, morphine has been listed on the WHO list of essential medicines (21st WHO Expert Committee, 2017). Activation of μ receptors induces analgesia via regulating the activity of Ca^2+^ and K^+^ channels. These modulations are mediated by Gßγ subunits, which directly interact with Ca_v_2.2 channels (Jeong & Ikeda, 2000), thereby decreasing Ca^2+^ influx and neuronal excitability as well as the release of pronociceptive transmitters (Tedford & Zamponi, 2006). The interaction of Gßγ subunits with G‐protein coupled inwardly rectifying (K_ir_3.X) channels causes K^+^ efflux, thereby causing postsynaptic hyperpolarization (Lüscher & Slesinger, 2010; Nockemann et al., 2013).

As they are expressed in the plasma membrane, GPCRs are exposed to changes in membrane potential. Several GPCRs have been previously reported to be modulated in their activity by the membrane potential. The ligands of most of the transmembrane receptors that have been characterized so far are of aminergic (Sahlholm, Marcellino, Nilsson, Fuxe, & Arhem, 2008; Sahlholm, Marcellino, Nilsson, Fuxe, & Århem, 2008; Sahlholm, Nilsson, Marcellino, Fuxe, & Arhem, 2012), purinergic (Gurung, Martinez‐Pinna, & Mahaut‐Smith, 2008; Martinez‐Pinna et al., 2005), glutamatergic (Ohana, Barchad, Parnas, & Parnas, 2006), lipidic (Martinez‐Pinna, Gurung, Mahaut‐Smith, & Morales, 2010) or cholinergic (Ben‐Chaim, Tour, Dascal, Parnas, & Parnas, 2003; Dekel, Priest, Parnas, Parnas, & Bezanilla, 2012; Kupchik et al., 2011; Parnas et al., 2005; Rinne, Mobarec, Mahaut‐Smith, Kolb, & Bünemann, 2015) nature. Although the first reports describing structures that may be part of the voltage‐sensing mechanism of certain receptors have appeared (Ben‐Chaim et al., 2006; Bezanilla, 2008; Rinne et al., 2015), a general mechanism underlying voltage sensitivity of GPCRs needs to be identified. So far, voltage sensitivity of GPCRs differs in magnitude and quality. Both agonist affinity (Ben‐Chaim et al., 2003; Ohana et al., 2006; Rinne et al., 2015; Rinne, Birk, & Bünemann, 2013; Sahlholm, Marcellino, Nilsson, Fuxe, & Arhem, 2008) and efficacy (Birk, Rinne, & Bünemann, 2015; Gurung et al., 2008; Rinne et al., 2013) can be modulated by voltage in a ligand‐specific way (Navarro‐Polanco et al., 2011; Rinne et al., 2013).

Due to the lack of non‐voltage‐sensitive receptor mutants that exhibit normal binding properties for their natural ligands, the physiological role of the voltage sensitivity of GPCRs is extremely difficult to study. Nevertheless, many GPCRs are expressed in the plasma membrane of highly excitable cells. A prominent example is the μ receptor for which we discovered robust voltage sensitivity as described in the current study. Neuropeptides such as enkephalins, endorphins and dynorphins, can activate μ receptors as endogenous agonists, whereas a variety of opioid analgesics, such as morphine or fentanyl, are clinically used for their pain‐relieving properties mediated via μ receptor activation (see Stein, 2016). Here, we not only focus on the general voltage sensitivity of μ receptors but also distinguish between effects of depolarization on alkaloid drugs (morphine), peptide substances ([D‐Ala^2^, N‐Me‐Phe^4^, Gly^5^‐ol]‐enkephalin; DAMGO or Met‐enkephalin; ME) and synthetic analgesics (fentanyl) and investigate downstream effects of voltage sensitivity. To show that the voltage sensitivity of responses of the μ receptors is present in native tissues, we also studied this sensitivity in locus coeruleus (LC) neurons in rat brain slices.

## 2 METHODS

### 2.1 Cell culture

HEK cells (HEK293T; CCLV Cat# CCLV‐RIE 1018, RRID:CVCL_0063) were cultured according to standard protocols (Vilardaga, Bünemann, Krasel, Castro, & Lohse, 2003) and transfected with Effectene transfection reagent (Qiagen) according to the manufacturer's protocol in 6‐cm dishes 2 days prior to experiments. The following plasmids were used: for G‐protein activation, wild‐type μ receptor (MOP‐wt): 0.5 μg, Gα_i_‐YFP or Gα_o_‐YFP: 1.0 μg, respectively, Gß_1_‐mTur2: 0.5 μg and Gγ_2_‐wt: 0.25 μg; for arrestin3 recruitment: MOP‐sYFP2: 0.7 μg, GRK2‐wt: 0.7 μg and Arr3‐mTur2: 0.7 μg; for GRK2 recruitment: MOP‐sYFP2: 0.6 μg, GRK2‐mTur2: 0.6 μg, Gα_i_‐wt: 0.7 μg, Gß_1_‐wt: 0.6 μg and Gγ_2_‐wt: 0.5 μg; note that for the bystander Förster Resonance Energy Transfer (FRET) experiment, an additional 0.5 μg of muscarinic M_3_ receptor were added to the GRK2 recruitment plasmids; and for K_ir_3.X current measurements, MOP‐wt: 0.3 μg, bicistonic plasmid expressing K_ir_3.1 and K_ir_3.4 subunits: 0.5 μg and pcDNA3‐eCFP: 0.2 μg. On the day before experiments, cells were split on poly‐l‐lysine coated coverslips.

### 2.2 FRET and electrophysiological measurements in HEK293T cells

As described before (Rinne et al., 2013), recordings of fluorescence can be made simultaneously with electrophysiological measurements (see Figure 4a) on an inverted Zeiss Axiovert microscope (Axiovert 135, Zeiss, Oberkochen, Germany). The microscope was equipped with an oil‐immersion objective (A‐plan 100×/1.25, Carl Zeiss), a Polychrome V light source and a dual‐emission photometry system (both TILL Photonics, Martinsried, Germany). FRET ratios were determined measuring CFP and YFP emission. Measurements were performed using single transfected cells which were excited with brief light flashes (F_430_, duration: 5 ms; frequency: 2.5 Hz at *λ* = 425 nm; excitation filter: ET 436/20, beam splitter: DCLP460; all beam splitters or filters were purchased from AHF [Tübingen, Germany] with a Polychrome V [TILL Photonics] light source). Collection of emitted donor fluorescence (*F*_480_) and acceptor fluorescence (*F*_535_) was performed with photodiodes (beam splitter: DCLP505; emission filters D480/40 and HQ535/30) and detected using photodiodes (TILL Photonics Dual Emission System). Digitalization was achieved using a computer interface (ITC16, HEKA Elektronik, Lamprecht, Germany). Recording of individual traces representing donor and acceptor emission and calculation of FRET ratio (*F*_535_/*F*_480_) were performed on a personal computer equipped with Patchmaster software (v2X52, HEKA Elektronik [Patchmaster, RRID:SCR_000034]) at 2.5 Hz.

During measurements, cells were superfused with either external buffer (NaCl 137 mM, CaCl_2_ 2 mM, KCl 5.4 mM, MgCl_2_ 1 mM, HEPES 10 mM; pH = 7.4) or agonist‐containing buffer solution using a pressurized superfusion system (ALA Scientific Instruments, Farmingdale, USA) which allows for rapid change of solutions. Borosilicate glass capillaries (GC150F‐10, Harvard Apparatus, Holliston, USA) were used to pull patch pipettes of 3‐ to 7‐MΩ resistance with a horizontal pipette puller (P87, Sutter Instruments, Novato, USA). Cells were patched in whole‐cell voltage‐clamp configuration, in which V_M_ was set to desired values using an EPC‐10 (HEKA) amplifier. Glass pipettes were filled with internal buffer (K^+^ aspartate 105 mM, KCl 40 mM, NaCl 5 mM, MgCl_2_ 7 mM, HEPES 20 mM, EGTA 10 mM, GTP 0.025 mM, Na^+^‐ATP 5 mM; pH = 7.2). K_ir_3.4 measurements in HEK293T cells were performed in whole‐cell voltage‐clamp configuration with inward or outward current recording in 1‐kHz sampling intervals. Cells were superfused with external buffer (as above) or a high K^+^ concentration containing buffer (as above, but with 140‐mM KCl and 2.4‐mM NaCl). Measurements in HEK293T cells were performed at room temperature.

### 2.3 Analysis of charge movements, deactivation kinetics and concentration‐dependent responses

Normalized values for the degree of receptor activation (*R*), reflected by arrestin3 recruitment dependent on V_M_, were fit to a single Boltzmann function (Figure 6b). Analysis was performed using GraphPad Prism 8 (GraphPad Software, La Jolla, USA [GraphPad Prism, RRID:SCR_002798]). The equation used for fitting was

$$R=R_{\min}+\frac{R_{\max}-R_{\min}}{1+\exp\left(\frac{V_{0.5}-V}{k}\right)},$$

where *R*_min_ and *R*_max_ are minimal and maximal response (i.e., μ receptor–arrestin3 interaction), *V* is the respective membrane potential, *V*_0.5_ is the voltage for half‐maximal effect on the observed interaction and *k* is the slope factor. In order to calculate the *z* factor (net charge movement upon change in V_M_ across the membrane), the following equation was applied as suggested by the software:

$$z=\frac{-26}{k}.$$

Determination of kinetics of receptor deactivation upon agonist withdrawal or repolarization were performed by fitting the FRET response of the receptor–arrestin3 interaction (indicated in Figure S6C) to a first‐order exponential decay. For determination of concentration–response curves, morphine‐induced FRET responses (measured as interaction of μ receptors and GRK2 at −90 and +30 mV) were normalized to maximum responses (FRET/FRET_10‐μM DAMGO_) and plotted against morphine concentration and fitted to a sigmoid curve. Fittings and calculations of *k*_off_ or EC_50_ values were performed using GraphPad Prism 8 (GraphPad Software).

### 2.4 Analysis of the possible contribution of bystander FRET, due to translocation to the plasma membrane

We used FRET to measure the direct GRK2 recruitment by μ receptors. However FRET responses can also be caused by interaction of GRK2 with endogenously expressed receptors in the crowded cell membrane, an indirect effect called “bystander FRET” (Clayton & Chattopadhyay, 2014) To assess the possible contribution of bystander FRET in our model system, we also measured FRET between MOP‐sYFP2 and GRK2‐mTur2 in the presence of overexpressed, unlabelled M_3_ receptors upon ACh stimulation. In this setting, which differed from the cellular setting for normal voltage‐clamp measurements through the overexpression of M_3_ receptors, we could observe bystander FRET of 0.26 ± 0.03 (mean ± SEM) of the DAMGO‐induced, μ receptor‐mediated maximum response (Figure S5C,D). Thus, we assume that the influence of bystander FRET for this assay should not be too large in cells, which are not additionally transfected with M_3_ receptors.

### 2.5 Brain slice preparation

All animal care and experimental procedures were performed in accordance with the UK Animals (Scientific Procedures) Act 1986, the European Communities Council Directive 1986 (86/609/EEC) and the University of Bath ethical review document. Animal studies are reported in compliance with the ARRIVE guidelines (Kilkenny, Browne, Cuthill, Emerson, & Altman, 2010) and with the recommendations made by the *British Journal of Pharmacology*.

Male Wistar rats (4–5 weeks old) (RRID:RGD_737929; originally purchased from Charles River and then bred at the University of Bath for >10 years) were housed up to four per cage under a 12‐h light–dark cycle (lights on at 7 a.m.) at a constant temperature of 21–23°C with ad libitum access to food and water. Rats were killed by decapitation under anaesthesia induced with ketamine (80 mg·kg^−1^) and xylazine (12 mg·kg^−1^). Brains were removed and submerged in ice‐cold cutting solution containing (in mM) 20 NaCl, 2.5 KCl, 0.5 CaCl_2_, 7 MgCl_2_, 1.25 NaH_2_PO_4_, 85 sucrose, 25 d‐glucose and 60 NaHCO_3_ and saturated with 95% O_2_/5% CO_2_. Horizontal brain slices (230 μm thick) containing the LC were prepared using a vibratome (DTK‐1000, Ted Pella, Redding, USA). Immediately upon cutting, slices were transferred to artificial CSF (aCSF) composed of (in mM) 126 NaCl, 2.5 KCl, 1.2 MgCl_2_, 2.4 CaCl_2_, 1.2 NaH_2_PO_4_, 11.1 d‐glucose, 21.4 NaHCO_3_ and 0.1 ascorbic acid; saturated with 95% O_2_/5% CO_2_ at 32°C; and left to equilibrate for at least 1 h prior to recording.

### 2.6 Whole‐cell patch‐clamp recordings in brain slices

Slices were submerged in a slice chamber (0.5 ml) mounted on a microscope stage (Scientifica, Uckfield, UK) and superfused (2.0–2.5 ml·min^−1^) with aCSF at 32°C. LC neurons were visualized using an upright microscope (BX51WI; Olympus, Southend‐on‐Sea, UK), and individual cell somata were cleaned by gentle flow of aCSF from a pipette. Whole‐cell patch‐clamp recordings in brain slices were made using glass pipettes (3–6 MΩ) filled with (in mM) 115 K‐gluconate, 10 NaCl, 2 MgCl_2_, 10 HEPES, 11 EGTA, 2 MgATP and 0.5 Na_2_GTP;pH 7.3; osmolarity, 270 mOsm. Recordings of whole‐cell currents were filtered at 1 kHz using an Axopatch 200B amplifier (Molecular Devices, Sunnyvale, CA) and analysed off‐line using WinEDR (University of Strathclyde, Glasgow, UK).

LC neurons were voltage‐clamped at −60 mV with correction for a −12‐mV liquid junction potential. Activation of μ receptors evoked K_ir_3.X currents. All drugs were applied in the superfusing solution at known concentrations and in the presence of tetrodotoxin (1 μM).

### 2.7 Data and statistical analysis

Data were analysed with OriginPro 9.1 (Originlab, Northampton, MA [Origin (RRID:SCR_014212)]) or GraphPad Prism 8 (GraphPad Software, La Jolla, CA). Measurements from G‐protein activation, arrestin3 and GRK2 recruitment which are prone to extensive photobleaching were corrected by subtracting a mono‐exponential decay function (for correction, see Figure S4A). Measurements from K_ir_3.X recordings (HEK293T cells) were smoothed using a low pass FFT filter with a cut‐off frequency of 125 Hz. Data represent single measurements or averaged values, shown as mean ± SEM. Group size is defined by *n* where in case of LC recordings, *n* defines the number of rats measured (one slice per rat) and in case of HEK cell measurements, *n* defines the number of cells measured. Group size was not estimated prospectively. Differences in group size (i.e., Figure 4e, morphine, and Figure 5b) result from additional investigations using same protocols for other purposes. If ANOVA was conducted on these groups, it was reassured that all *n*'s were included in group comparisons. Statistical analysis was only performed when group sizes in each group were *n* ≥ 5. Statistical comparisons were performed with a paired Student's *t*‐test, two‐tailed unpaired *t*‐test or one‐way ANOVA with Tukey's multiple comparison as indicated, and difference was considered significant, if *P* < 0.05. Before conducting ANOVA post hoc tests, variance homogeneity and significance of *F* were confirmed. Normality of data distribution was tested using a Shapiro–Wilk test, and parametrical statistical tests were only carried out if normality was confirmed. Concentration–response curves were fitted using a least‐squares fit. Models were compared using an extra‐sum of squares *F*‐test. Cells and slices were selected for data collection in a randomized fashion. Both data collection and data evaluation were performed unblinded for practical reasons. This is mainly due to the fact that the experimenter has to apply agonists by himself and also set the V_M_ protocol. However, for evaluation of data, the same criteria were applied for agonist with regard to bleach correction or statistical analysis. The data and statistical analysis comply with the recommendations of the *British Journal of Pharmacology* on experimental design and analysis in pharmacology (Curtis et al., 2018).

### 2.8 Normalization

Individual responses were normalized to maximum responses of the same cell within the same measurement protocol in most cases. In case of LC recordings, where no application of a maximum concentration was applied due to long washout times or to avoid desensitization, responses to agonists at −80 or −40 mV were normalized to responses to the same ligand at −60 mV. For fitting of data with a Boltzmann function (for more detail, see Section 2.3), data were normalized to responses of the cell to morphine at 0 mV. Normalization of current responses in HEK293T cells is explained in more detail below.

### 2.9 Normalization of K_ir_3.X current responses evoked by non‐saturating opioid concentrations (HEK293T cells)

We compared K_ir_3.X currents evoked by non‐saturating concentrations of different opioids (morphine, DAMGO, ME and fentanyl) to maximum responses evoked by a saturating concentration of opioid (DAMGO, 30 nM). Due to a functional receptor reserve (Connor, Osborne, & Christie, 2004), voltage‐dependent activation of the μ receptors would be concealed under saturating concentration of any of the opioids tested. To prevent saturation, for K_ir_3.X measurements of agonists which showed voltage‐dependent activation upon depolarization (i.e., morphine and DAMGO) in HEK293T cells, only those cells were taken into evaluation which gave an opioid response at holding potential (i.e., −90 mV/−50 mV) below 0.3‐fold of maximum DAMGO response. In case of high K^+^ measurements, this led to exclusion of eight cells under morphine application, and for DAMGO, five cells had to be excluded due to high initial responses that might run into saturation under further depolarization‐induced activation. In the morphine measurements under physiological K^+^ concentration, no cell had to be excluded for that reason. With morphine, we used higher concentrations of morphine (22.5 nM instead of 4.5 nM) when we measured outward K^+^ currents. We assumed differences in receptor expression during that time. The difference in ligand concentrations used was of minor importance to our conclusions, as we were focused on the proportional increase of responses, which were normalized to a maximum response. To prevent saturation and to enable observation of depolarization‐induced decrease in K_ir_3.X currents, for fentanyl (which in initial trials had shown to be deactivated upon membrane depolarization), we chose to include cells which gave up to 0.9‐fold of maximum response at −90 mV. This led to exclusion of two cells, which gave higher responses in non‐depolarized condition. For evaluation of the proportional opioid response, we always calculated the mean current response during the last second before agonist withdrawal or repolarization to holding potential, respectively. Furthermore, depolarization to 0 mV caused a short current spike in the outward direction, which returned to baseline within 10 s and was therefore omitted from the calculation of current response amplitudes.

To speed up washout procedures, for measurements of K_ir_3.X currents in HEK293T cells, we sought to use the lowest concentration possible to generate a maximum response (DAMGO, 30 nM). This concentration was determined by a concentration–response curve as shown in Figure S1C and differed from concentrations that were needed to obtain a maximum response in other assays (Figures S4C and 6f). The concentration of DAMGO that was necessary to obtain a maximum K_ir_3.X current response also differed from other studies investigating K_ir_3.X currents (Yudin & Rohacs, 2019) which may be due to differences in experimental design (i.e., co‐transfection of heterotrimeric G‐protein).

### 2.10 Ramps

We showed current–voltage relationships of GIRK currents (inward and outward) in HEK293T cells by plotting background (evoked in absence of agonist)‐substracted agonist‐induced currents (evoked by 30 nM DAMGO) against respective V_M_. For ramps, cells were kept at −120 mV for 200 ms and subsequently depolarized to +60 mV within 400 ms while currents were recorded with a sample interval of 1 kHz.

### 2.11 Cloning of plasmids

Gß_1_‐mTur2 has been generated by exchange of the Gß_1_‐Cer (Frank, Thümer, Lohse, & Bünemann, 2005) fluorophore with mTurquoise2.

### 2.12 Materials

For HEK293T cell experiments, morphine hydrochloride was purchased from Merck, DAMGO acetate salt, [Met^5^]enkephalin acetate salt and fentanyl citrate were purchased from Sigma Aldrich. For LC slice experiments, all reagents were purchased from Sigma Aldrich, except [Met^5^]‐enkephalin, DAMGO (Bachem), morphine sulfate (MacFarlan Smith), naloxone and tetrodotoxin (HelloBio).

### 2.13 Nomenclature of targets and ligands

Key protein targets and ligands in this article are hyperlinked to corresponding entries in http://www.guidetopharmacology.org, the common portal for data from the IUPHAR/BPS Guide to PHARMACOLOGY (Harding et al., 2018), and are permanently archived in the Concise Guide to PHARMACOLOGY 2015/16 (Alexander, Christopoulos et al., 2019; Alexander, Mathie et al., 2019).

## 3 RESULTS

### 3.1 Membrane potential regulates μ receptor‐induced K_ir_3.X currents in HEK293T cells

The potential voltage sensitivity of μ receptors was first investigated by recording μ receptor‐induced G‐protein coupled K_ir_3.X current responses in response to different opioids and holding potentials in HEK293T cells. Signalling by μ receptors, via Gßγ subunit‐mediated activation of K_ir_3.X channels, is involved in therapeutically desired antinociception (Corbett et al., 2006).

**FIGURE 1:**
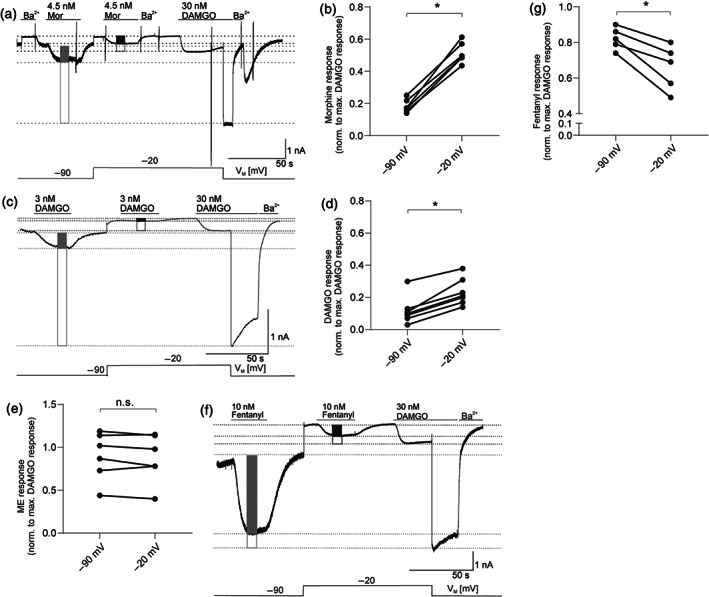
Membrane potential regulates μ receptor‐induced K_ir_3.X currents in HEK293T cells. (a) Representative recording (out of *n* = 6) of inward K^+^ currents in HEK293T cells expressing wild‐type μ receptors (MOP‐wt) and K_ir_3.X channels is shown. K_ir_3.X currents were evoked by 4.5 nM morphine (Mor) and 30 nM DAMGO and measured at −90 and −20 mV (voltage protocol is depicted at the bottom; note that depolarization to −20 mV shifts both basal and active currents due to the current–voltage relationship [see Figure S1] of the channel). Levels that were used for calculation of maximum responses are shown as light grey line (−90 mV) or dark grey line (−20 mV), and amplitude height is shown by boxes (morphine response: filled box; maximum DAMGO response: empty box). (b) K_ir_3.X current responses evoked by non‐saturating morphine concentrations were normalized to the maximum response (evoked by a saturating DAMGO concentration) at respective membrane potentials (*n* = 6, for further explanation of evaluation, see Section 2). (c) Representative recording (out of *n* = 7) of inward K^+^ currents. K_ir_3.X currents were evoked by 3 nM morphine and 30 nM DAMGO and measured and evaluated as explained in (a) (non‐saturating DAMGO response: filled box; saturating response: empty box). (d) K_ir_3.X current responses evoked by non‐saturating DAMGO concentrations were normalized to the maximum response (evoked by a saturating DAMGO concentration) at respective membrane potentials (*n* = 7, for further explanation of evaluation, see Section 2). Responses at −90 and −20 mV were compared in the same recording. *P < 0.05, significantly different as indicated; paired two‐sample Wilcoxon test. (e) K_ir_3.X current responses evoked by non‐saturating Met‐enkephalin concentrations were normalized to the maximum response (evoked by a saturating DAMGO concentration) at respective membrane potentials (*n* = 6, for further explanation of evaluation, see Section 2). (f) Representative recording (out of *n* = 5) of inward K^+^ currents. K_ir_3.X currents were evoked by 10 nM fentanyl and 30 nM DAMGO and measured at −90 and −20 mV and measured and evaluated as explained in (a) (fentanyl response: filled box; saturating DAMGO response: empty box). (g) K_ir_3.X current responses evoked by non‐saturating fentanyl concentrations were normalized to the maximum response (evoked by a saturating DAMGO concentration) at respective membrane potentials (*n* = 5, for further explanation of evaluation, see Section 2). (a, c, f) K_ir_3.X channels were blocked with barium (500 μM) at several time points as indicated. (b, e, g) As responses at −90 and −20 mV were compared in the same recording, statistics were performed using a paired, two‐tailed *t*‐test (n.s.: *P* > 0.05, *P < 0.05)

In high K^+^ buffer (see Section 2), the K_ir_3.X current–voltage relationship is linear when K^+^‐flux is inward (Figure S1B). This setting was used to study voltage‐mediated changes in μ receptor‐activated K_ir_3.X currents over a broad range of negative membrane potentials. Given the functional receptor reserve (Connor et al., 2004), which would conceal voltage‐dependent activation under saturating concentration of any opioid, we applied a non‐receptor‐saturating concentration of morphine (4.5 nM) and compared those responses to maximum responses which were obtained by application of a saturating DAMGO concentration (30 nM, Figure S1C,D, Section 2). At −90 mV, the proportional morphine‐evoked response was 18% (±2%) of maximum currents which increased at −20 mV up to 51% (±3%), corresponding to a 2.8‐fold increase in the relative response (Figure 1a,b). Ba^2+^ sensitivity of K_ir_3.X currents was confirmed during measurements, as indicated (Figure 1a,c,f and S1C).

We next tested for the voltage sensitivity of μ receptor signalling induced by opioids with different structural properties. K_ir_3.X currents in response to a non‐saturating concentration (3 nM) of DAMGO (Figure S1C), which is a synthetically modified peptide μ receptor agonist (Handa et al., 1981), revealed a minor voltage sensitivity of DAMGO (Figure 1c,d): the fractional current response of 12% (±3%) at holding potential of −90 mV increased 1.9 fold to 23% (±3%) upon depolarization to −20 mV. We also compared ME‐mediated K_ir_3.X currents between different holding potentials and found that this peptide ligand showed no voltage‐sensitive behaviour at all (Figure 1e). When we characterized fentanyl that differs structurally with its anilinopiperidine structure from both the alkaloid analgesics and the peptide natural ligands, we observed a remarkable result. In contrast to morphine, fentanyl‐mediated currents were decreased when the membrane was depolarized (from 82% at −90 mV [±3%] to 66% at −20 mV [±6%] of maximum response), reflecting a decrease in the relative current response by 20% (Figure 1f,g).

### 3.2 The observed voltage sensitivity occurs also in physiological K^+^ concentrations

**FIGURE 2:**
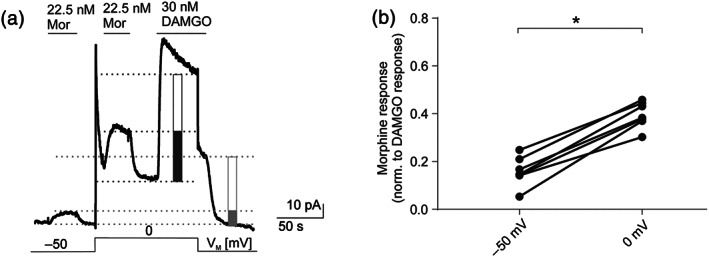
The observed voltage sensitivity occurs also in physiological K^+^ concentrations*.* (a) Representative recording (*n* = 7) of outward K^+^ currents measured in HEK293T cells expressing wild‐type μ receptors (MOP‐wt) and K_ir_3.4 channels. K_ir_3.X currents were evoked by 22.5 nM morphine (Mor; non‐saturating, also see Section 2) and 30 nM DAMGO (saturating) and measured at −50 and 0 mV. Levels that were used for calculation of maximum responses are depicted as light grey line (−50 mV) or dark grey line (0 mV), and amplitude height is shown by boxes (morphine response: filled box; saturating DAMGO response: empty box). Voltage protocol is depicted at the bottom; note that depolarization steps shift both basal and active currents due to the current–voltage relationship of the channel (see Figure S2). (b) K_ir_3.X current responses evoked by non‐saturating morphine concentrations were normalized to the maximum DAMGO response at respective membrane potentials (*n* = 7, for further explanation of evaluation, see Section 2). Responses at −50 and 0 mV were compared in the same recording. *P < 0.05, significantly different as indicated; paired *t*‐test

Under physiological conditions, K^+^ flows in an outward direction and K_ir_3.X currents are therefore smaller due to their property of inward rectification (Figure S2A,B). Therefore, outward morphine‐induced K_ir_3.X current responses could only be compared between −50 and 0 mV and were displayed in relation to a maximum response (induced by 30 nM DAMGO, see Figure S1C,D, Section 2) at respective membrane potentials (Figure 2a). At V_M_ (−50 mV), morphine‐evoked responses were increased upon depolarization from −50 to 0 mV from 16% ± 2% to 40% ± 2% of the respective maximal response, reflecting a 2.5‐fold increase in relative current response (Figure 2b). Therefore, the pronounced voltage sensitivity of K_ir_3.X currents evoked by morphine was seen with both inward and outward flows of K^+^.

### 3.3 Voltage sensitivity of morphine‐induced K_ir_3.X currents can be detected in physiological tissue

The absence of detectable voltage‐dependence of responses to ME (see Figure 1e) made this agonist ideal as a reference compound for studying voltage‐induced effects of morphine in brain slices. The LC is a well‐established tissue to characterize μ receptor‐evoked K_ir_3.X currents due to the fact that it expresses μ receptors without co‐expression of δ or κ receptors (North & Williams, 1985). We therefore recorded μ receptor‐induced K_ir_3.X currents in LC neurons using brain slices from male Wistar rats (4–5 weeks).

**FIGURE 3:**
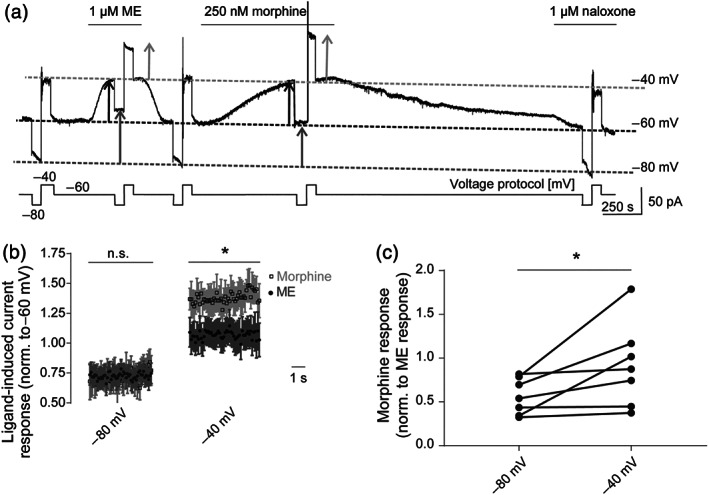
Voltage sensitivity of morphine‐induced K_ir_3.X currents can be detected in physiological tissue. (a) Representative recording of outward K^+^ currents in LC neurons. K_ir_3.X currents were evoked by non‐saturating concentrations of morphine (Mor; 250 nM) or Met‐enkephalin (ME; 1 μM). During measurements, cells were kept at −60 mV and briefly clamped to −80 and −40 mV in turn (voltage protocol is depicted below the current trace). Initially, baseline current responses at each membrane potential were determined by voltage steps in control aCSF, followed by responses in the presence of ME and then morphine. To accelerate washout of morphine, naloxone (1 μM) was applied afterwards. Dotted lines depict the baseline currents at each holding potential (dark grey: −80 mV; black: −60 mV; light grey: −40 mV). The magnitude of agonist‐induced currents at each holding potential is shown as arrows (dark grey: −80 mV; black: −60 mV; light grey: −40 mV). (b) Average (mean ± SEM) time course of current responses following application of non‐saturating concentrations of morphine or Met‐enkephalin. For each agonist, individual responses at −80 and −40 mV were normalized to current responses of the respective ligand at −60 mV. *P < 0.05, significantly differences as indicated, between ME and morphine responses at −40 mV; *n* = 7. (c) K_ir_3.X currents (as shown in Figure 3a) evoked by a non‐saturating morphine concentration were normalized to current responses evoked by a non‐saturating Met‐enkephalin concentration at respective membrane potentials, and the relative voltage‐dependent effect induced by morphine was compared at −80 and −40 mV, without normalization to individual cell responsiveness at −60 mV. *P < 0.05, significantly different as indicated; paired *t*‐test; *n* = 7

Due to limitations of electrophysiological recordings from LC neurons such as spontaneous neuronal firing (North & Williams, 1985), the maximum possible achievable membrane depolarization was −40 mV in these neurons. The holding potential was −60 mV and was stepped to −80 and −40 mV. The K_ir_3.X currents induced by μ receptor agonists were recorded at each potential (Figure 3a). Equi‐effective concentrations of ME and morphine (1 and 0.25 μM, respectively) were applied which resulted in approximately 50% of the maximum μ receptor response. Agonist‐evoked current responses were then calculated by subtraction of the baseline current at each membrane potential. As washout of morphine was very slow in this tissue, we applied naloxone (1 μM) after morphine application to be able to determine baseline current also at the end of the experiment. In consideration of the large interindividual differences in amplitude heights, we normalized the calculated current responses at −80 and −40 mV to the respective agonist‐induced response at −60 mV. At −80 mV, no significant difference in current responses of morphine and ME could be observed but, at ‐40mV, the spread between ME and morphine responses indicated a significantly higher current response to morphine, compared with that to ME (Figure 3b). We also evaluated the data by normalizing the morphine‐induced current response at −80 and −40 mV to the respective ME responses. Again, in each cell, a higher morphine‐evoked current response in proportion to ME could be observed at −40 mV compared to that at −80 mV (Figure 3c). Note that in some cells, differences in responsiveness were not very pronounced, which might be due to saturation in individual cells. Even using this method, where interindividual differences were still evident, the voltage‐sensitive increase of morphine‐mediated current was still significant when compared with ME‐evoked currents.

For control purposes, two repeated ME applications in the same recording were compared to exclude some general changes in K_ir_3.X currents over the time course of the protocol (Figure S3A,B). There was no increase in voltage sensitivity of ME on the second application compared with the first; indeed, there was a small but significant decrease. Thus, the observed relative increase in morphine‐evoked K_ir_3.X currents (Figure 3a–c) is not caused by protocol (time)‐dependent increases of K_ir_3.X current amplitudes. In a second set of control measurements, we performed single applications of morphine, ME and DAMGO, in separate recordings. We compared current amplitude changes on depolarization from −80 to −60 mV and from −60 to −40 mV for each agonist. While the increase in current amplitude, which is caused by the current–voltage relationship, was increased to a similar extent for all three ligands upon depolarization from −80 to −60 mV, we saw a significant increase of morphine‐evoked currents upon depolarization from −60 to −40 mV (Figure S3C,D), which was in accordance with our earlier observations from the ME–morphine protocol (Figure 3a).

### 3.4 Ligand‐specific voltage sensitivity occurs at the level of μ receptor activation and is transduced to G‐protein activation

**FIGURE 4:**
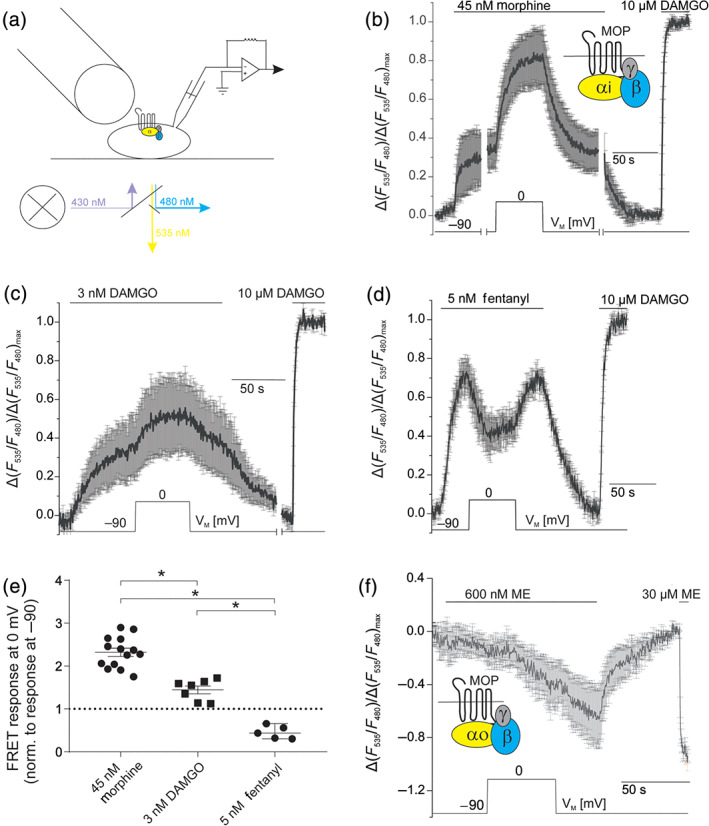
Ligand‐specific voltage sensitivity occurs at the level of μ receptor activation and is transduced to G‐protein activation. (a) Diagram of the configuration of FRET‐based assays under whole‐cell voltage‐clamp conditions. Cells were transfected with μ receptors (MOP), Gα_i_‐YFP, Gß1‐mTur2 and Gγ2 and subjected to dual‐emission fluorescence microscopy under continuous superfusion (as indicated) with buffer or agonist‐containing buffer. (b) Average (mean ± SEM) of FRET recordings (*n* = 6 of 14 similar measurements, for which the same time course in the protocol was applied) plotted as relative agonist‐evoked alterations in the YFP/mTur2 emission ratio. Morphine‐ or DAMGO‐induced G_i_‐protein activation is reflected as an increase in FRET measured between YFP‐labelled Gα_i_ and mTur2‐labelled Gß_1_, as shown previously (Bünemann, Frank, & Lohse, 2003). (c) Average (mean ± SEM; *n* = 7) of FRET traces showing G_i_‐protein activation evoked by DAMGO (3 nM; non‐saturating) (McPherson et al., 2010). (d) Average (mean ± SEM; *n* = 5) of FRET traces showing G_i_‐protein activation evoked by fentanyl (5 nM; non‐saturating). (b–d) Maximum responses were determined by application of a saturating DAMGO concentration (10 μM) at the end of experiments. (e) Comparison of alterations in agonist‐induced FRET changes due to depolarization from −90 to 0 mV for morphine (mean ± SEM; *n* = 14), DAMGO (mean ± SEM, *n* = 7) and fentanyl (mean ± SEM, *n* = 5). *P < 0.05, significantly different as indicated; one‐way ANOVA with Tukey's multiple comparison test; dotted line indicates voltage insensitivity, as no change in signalling upon depolarization occurs. (f) Average (mean ± SEM; *n* = 5) of FRET recordings plotted as relative agonist evoked alterations in the YFP/mTur2 emission ratio. Met‐enkephalin‐induced G_o_‐protein activation is reflected as a decrease in FRET. Upon depolarization from V_M_ (−90 mV) to 0 mV, no change in G‐protein activation occurred

As recordings of single cell FRET‐based assays under conditions of whole‐cell voltage‐clamp (Figure 4a) are not limited by the reversal potential of K^+^, they allow for more thorough investigation of voltage sensitivity including analysis of a manifold spectrum of signalling interactions.

To study voltage sensitivity of ligand‐mediated G‐protein signalling, we transfected HEK293T cells with wild‐type μ receptors (MOP‐wt), Gα_i_1(91)‐YFP, mTurq‐Gß_1_ and Gγ_2_, a reporter system that indicates G_i_‐protein activation by an increase in FRET (Bünemann et al., 2003). Due to a functional receptor reserve (Connor et al., 2004), non‐saturating concentrations of each of the μ receptor agonists were applied. Maximal activation of G‐proteins was always induced by application of 10 μM DAMGO (saturating concentration) at the end of the experiment. Morphine (45 nM) induced a slow and moderate increase in FRET (Figure 4b), which increased substantially (~2.3‐fold) upon depolarization from −90 to 0 mV, a robust increase in FRET, reflecting that G_i_‐protein activation was observed (Figure 4b,e). To avoid photobleaching artefacts, all FRET recordings were corrected for a mono‐exponential function (Figure S4A). Depolarization in the absence of agonist (Figure S4B) led to no G‐protein activation reflecting no μ receptor activation.

To assess comparability of measurements of K_ir_3.4 currents and G‐protein activation in HEK293T cells, we conducted the same protocol in both assays (Figures 2a and S4C). As observed in K_ir_3.4 current recordings, we found a very similar enhancement of morphine response upon depolarization (Figure S4C,D).

We next compared voltage sensitivity of μ receptor‐mediated G_i_ activation following application of structurally distinct ligands. Application of non‐saturating concentration (3 nM) of the peptide ligand DAMGO led to a weak G_i_ activation at −90 mV which was increased 1.4‐fold by depolarization to 0 mV (Figure 4c,e). A similar voltage dependence of DAMGO‐induced G_o_‐protein activation was confirmed making use of a FRET assay for G_o_‐protein activation (Frank et al., 2005) that reflects G‐protein activation by a decrease in FRET (Figure S4E). As in K_ir_3.X current recordings, the voltage dependence of G‐protein activation by fentanyl was the opposite to that seen with morphine, where G_i_‐protein activation by a non‐saturating concentration of fentanyl (5 nM) was reduced ~0.45‐fold at 0 mV compared with at −90 mV (Figure 4d,e). Statistical comparison of voltage‐induced changes in G‐protein activation upon application of the different agonists showed that morphine, DAMGO and fentanyl each were regulated by V_M_ to a different extent or in a different direction (Figure 4e). Also, as in K_ir_3.X current recordings, the effects of ME were not voltage sensitive as depolarization upon application of a sub‐saturating ME concentration (600 nM) did not change G_o_ activation (Figure 4f).

### 3.5 Voltage dependence of μ receptor signalling is by modulation of efficacy, as seen with GRK2 recruitment

The observed voltage‐dependent modulation of agonist‐evoked μ receptor signalling could be due to increased agonist affinity to the receptor or increased intrinsic efficacy of the ligand at the μ receptor. In order to address this question, we recorded a direct agonist‐induced receptor–effector interaction (Lohse, Vilardaga, & Bünemann, 2003), specifically GRK2–receptor and arrestin–receptor interactions. Evidence from the literature suggests a low potency of morphine for inducing GRK2 recruitment (Miess et al., 2018), arrestin binding (McPherson et al., 2010) and endocytosis (Arttamangkul, Quillinan, Low, Von Zastrow, & Pintar, 2008; Celver, Xu, Jin, Lowe, & Chavkin, 2004; Whistler, Chuang, Chu, Jan, & von Zastrow, 1999), and the ability of a μ receptor ligand to recruit GRK2 and arrestin3 is strongly correlated with the agonist's intrinsic efficacy at the receptor (McPherson et al., 2010). We therefore examined the influence of the membrane potential on the direct interaction of the ligand‐activated μ receptors with GRK2, which is the initiating step of the signalling cascade of arrestin3 (Ferguson, 2001; Krasel, Bünemann, Lorenz, & Lohse, 2005).

**FIGURE 5:**
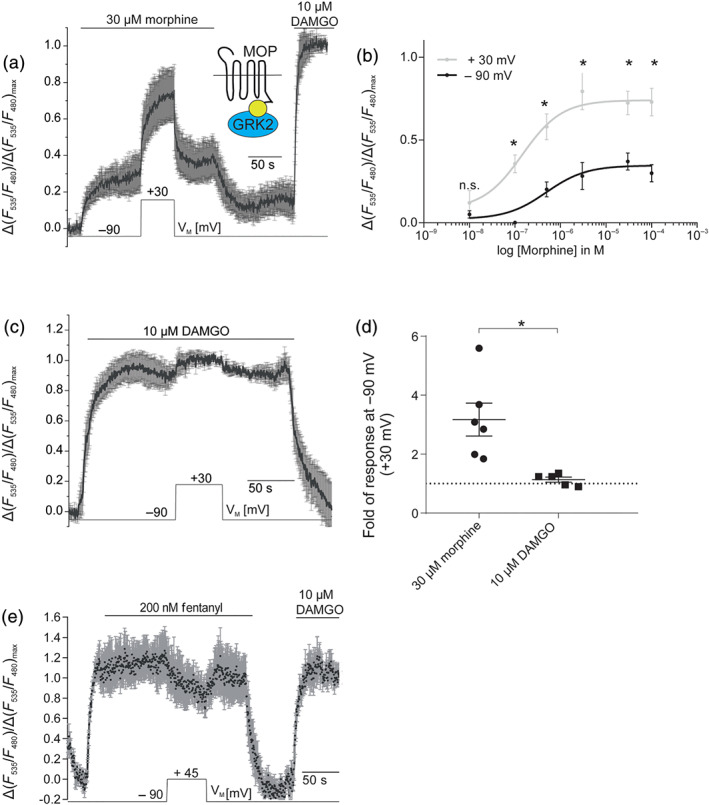
Efficacy of analgesics to recruit GRK2 is modulated by voltage. (a) Average (mean ± SEM; *n* = 6) of FRET recordings plotted as relative agonist evoked alterations in the sYFP2/mTur2 emission ratio. Morphine‐ or DAMGO‐induced GRK2 recruitment is reflected as an increase in FRET measured between sYFP2‐labelled μ receptors and mTur2‐labelled GRK2, as shown previously (Miess et al., 2018). For single emission traces, see Figure S4B. Depolarization from −90 to +30 mV reveals a robust enhancement of GRK2 recruitment to μ receptors upon morphine application. (b) Concentration–response curve was plotted based on averaged data collected as described in (a) at −90 and +30 mV at various concentrations of morphine (*n* = 5–16 per data point). *P < 0.05, significantly differences between data points measured at +30 mV compared with −90 mV at all concentrations except for 10 nM morphine; paired Student's *t*‐tests with Welch's correction. (c) Average (mean ± SEM; *n* = 5) of FRET recordings of μ receptor–GRK2 interaction upon application of 10 μM DAMGO. Cells were depolarized from −90 to +30 mV. (d) Summary of changes in μ receptor–GRK2 interaction upon depolarization from −90 to +30 mV under morphine or DAMGO application; responses were normalized to DAMGO response at 90 mV. *P < 0.05; unpaired *t*‐test; *n* = 5–6. Dotted line indicates voltage insensitivity as no change in signalling occurs upon depolarization. (e) Average (mean ± SEM; *n* = 5) of FRET recordings of μ receptor–GRK2 interaction upon application of 200 nM fentanyl. Cells were depolarized from −90 to +45 mV. (a, c, e) Maximum responses were determined upon application of DAMGO at −90 mV

We transfected HEK293T cells with MOP‐sYFP2, GRK2‐mTur2 and unlabelled heterotrimeric G_i_ proteins (Miess et al., 2018). Agonist‐induced GRK2 recruitment to the μ receptors was observed by an increase in FRET (Figures 5a and S5A,B) which was specific (Figure S5C,D) (Clayton & Chattopadhyay, 2014; King, Sarabipour, Byrne, Leahy, & Hristova, 2014). At −90 mV, a saturating morphine concentration (30 μM) led only to minor GRK2 recruitment to the activated receptor. However, this recruitment was increased from 30% (±9%) to 73% (±13%), when the membrane was depolarized from −90 to +30 mV (Figure 5a), suggesting a change in ligand efficacy in GRK2 recruitment. To further test this hypothesis, we measured concentration–response relationships for morphine at both −90 and +30 mV and found that the EC_50_ values obtained at the different holding potentials were not significantly different (EC_50, −90 mV_ = 0.49 ± 0.37 μM; EC_50, +30 mV_ = 0.15 ± 0.1 μM) whereas the normalized morphine‐evoked GRK2 recruitment was increased significantly upon depolarization (in all concentrations tested, except for 10 nM; Figure 5b). As observed in the G‐protein signalling assays, there was only a small voltage dependence of DAMGO‐induced GRK2 recruitment (12% ± 2% increase at +30 mV compared with at −90 mV; Figure 5c). The depolarization‐induced changes in the GRK2–μ receptor interaction were significantly larger for morphine compared to DAMGO (Figure 5d). In contrast to DAMGO and morphine, the analgesic fentanyl exhibited a slow but substantial decrease (−23% change ±10%) in GRK2 recruitment upon depolarization to +45 mV, which was reversible (Figure 5e). Again, this effect is similar to that observed with both fentanyl‐induced G‐protein activation and K_ir_3.X currents.

### 3.6 Voltage‐dependent efficacy modulation translates into altered arrestin3 recruitment

**FIGURE 6:**
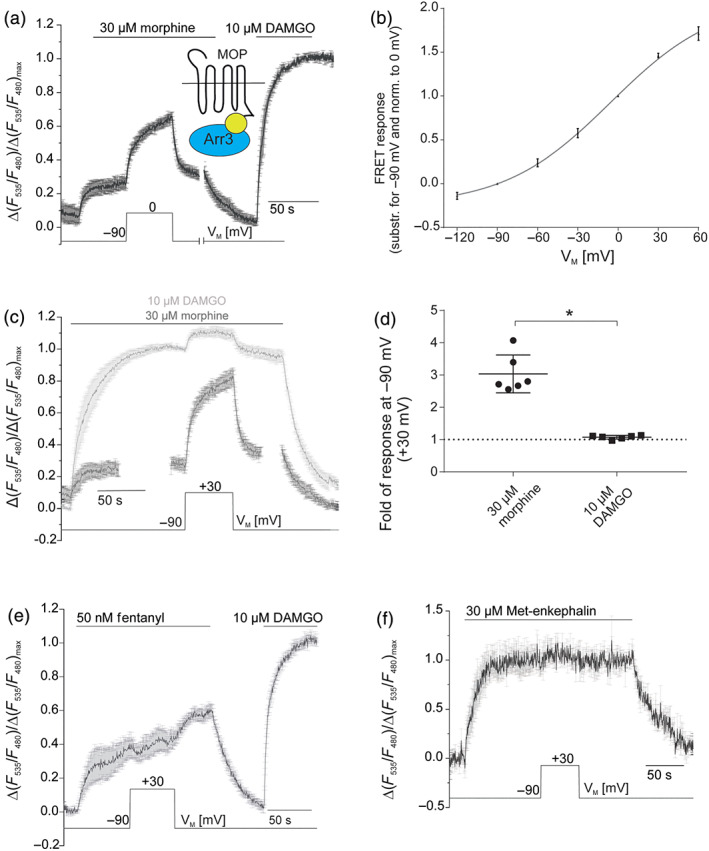
Voltage‐dependent efficacy modulation translates into altered arrestin3 recruitment. (a) Average (mean ± SEM; *n* = 6) of FRET recordings plotted as relative agonist evoked alterations in the sYFP2/mTur2 emission ratio. Morphine‐ or DAMGO‐induced arrestin3 recruitment is reflected as an increase in FRET measured between sYFP2‐labelled μ receptors and mTur2‐labelled arrestin3, as shown previously (McPherson et al., 2010). For single emission traces, see Figure S6A. (b) Voltage dependence of morphine‐induced (30 μM, saturating concentration) arrestin recruitment was determined by clamping the membrane from −90 mV to different test potentials and plotting voltage‐induced alterations in FRET relative to those obtained at 0 mV. Data were fitted to a Boltzmann function (*n* = 5–6 for different depolarization steps, *R* ^2^ = 0.99) giving rise to a V_0.5_ = −6 mV and corresponding *z*‐factor of 0.66. (c) Average (mean ± SEM; *n* = 6) of μ receptor–arrestin interaction upon application of 10 μM DAMGO or 30 μM morphine. Cells were depolarized from −90 to +30 mV. (a, c) Maximum responses were determined upon application of DAMGO (10 μM) at −90 mV. (d) Summary of changes in μ receptor–arrestin3 interaction upon depolarization from −90 to +30 mV, following morphine or DAMGO application. *P < 0.05; significantly different as indicated; unpaired *t*‐test; *n* = 6). Dotted line indicates voltage insensitivity as no change in signalling occurs upon depolarization. (e) Average (mean ± SEM; *n* = 5) of μ receptor–arrestin interaction upon application of 50 nM fentanyl, sub‐saturating concentration. Cells were depolarized from −90 to +30 mV. (f) Average (mean ± SEM; *n* = 5) of μ receptor–arrestin interaction upon application of 30 μM Met‐enkephalin, saturating concentration. Cells were depolarized from −90 to +30 mV

We further tested whether these voltage‐dependent effects could also be observed at the level of arrestin3 recruitment to the μ receptor. As previously shown (McPherson et al., 2010), morphine is less effective at recruiting arrestin3 compared with higher efficacy agonists such as the peptide ligand DAMGO or the anilinopiperidine fentanyl. Accordingly, HEK293T cells transfected with C‐terminally sYFP2‐labelled μ receptors and arrestin3‐mTur2 as well as GRK2‐wild‐type (McPherson et al., 2010) exhibited only a moderate increase in FRET upon exposure to a receptor‐saturating concentration of morphine (30 μM) compared to DAMGO (10 μM) at −90 mV (28% ± 3% of DAMGO response, Figure 6a). However, depolarization resulted in a rapid, robust and reversible increase (up to 65% ± 0.3%) in FRET in the presence of morphine (Figures 6a and S6A), indicating a voltage‐induced alteration in the efficacy of morphine. Ligand‐independent effects of V_M_ on receptor–arrestin3 interaction were not observed (Figure S6B).

We further analysed the voltage range which modulated the μ receptor–arrestin3 interaction induced by morphine, by fitting a Boltzmann function to the data (Figure 6b), which revealed a half‐maximal receptor activation at V_0.5_ = −6 mV and a *z*‐factor of 0.66. Furthermore, the kinetics of repolarization‐induced or agonist‐washout‐induced dissociation of receptor–arrestin3 complexes were significantly (twofold) faster upon repolarization, compared with agonist washout (Figure S6C–E), again suggesting a change in efficacy as the main cause of voltage‐induced changes in morphine‐evoked μ receptor signalling. Statistical comparison of depolarization‐induced increases of arrestin3 recruitment showed significantly higher voltage dependence with morphine (3.0‐fold ± 0.24) compared to DAMGO (1.1‐fold ± 0.02) (Figure 6c,d).

Similar to GRK2 recruitment, G‐protein activation and K_ir_3.X currents, the extent of voltage sensitivity of DAMGO‐induced arrestin3 recruitment was negligible (Figure 6c) and was not detectable for saturating concentrations of ME (Figure S6F) and fentanyl (Figure S6G).

Using non‐saturating concentrations of DAMGO (50 nM, Figure S6F) or fentanyl (50 nM, Figure 6e), we found that depolarization‐induced enhancement of μ receptor–arrestin3 recruitment was significantly less for DAMGO (1.3‐fold ± 0.03) compared with morphine (3.0‐fold ± 0.24) whereas alterations in the presence of fentanyl were again negligible (0.92‐fold ± 0.06) (Figure S6H).

## 4 DISCUSSION

We have demonstrated that the membrane potential has a considerable effect on the ability of opioid analgesics to activate μ receptors. This voltage sensitivity is an intrinsic property of the μ receptor, which is highly dependent on the ligand and is reflected in effects on downstream signalling such as G‐protein and K_ir_3.X channel activation as well as GRK2 binding and arrestin3 recruitment. Importantly, we confirmed the existence of the voltage sensitivity of μ receptors in LC neurons, measured in rat brain slices.

### 4.1 Voltage sensitivity is ligand specific

Voltage‐induced effects differed substantially between the alkaloid agonist morphine, the anilinopiperidine analgesic fentanyl and the peptide agonists DAMGO and ME. While morphine showed a 3.0‐fold depolarization‐induced increase (upon depolarization to +30 mV) in activity at the μ receptor (Figures 5a,b,d and 6a,b,d), DAMGO showed an only 1.1‐fold increase in activity upon depolarization (Figures 5c,d and 6c,d). Responses to the endogenous peptide μ receptor‐agonist ME were not altered by voltage to a detectable degree, as neither ME‐induced activation of G_o_ proteins and K_ir_3.X currents nor ME‐induced arrestin3 recruitment was sensitive to depolarization (Figures 1e, S3D, S4F and 6F). In contrast to morphine, for fentanyl, we saw a clear decrease of μ receptor‐mediated signalling upon depolarization in K_ir_3.X current activation, G_i_‐protein activation and GRK2 recruitment to the receptor (Figures 1f,g, 4d,e and 5e). Based on previous studies, we know that voltage can have an effect on both affinity (Ben‐Chaim et al., 2003; Ohana et al., 2006; Rinne et al., 2013, 2015; Sahlholm, Marcellino, Nilsson, Fuxe, & Arhem, 2008) and efficacy (Gurung et al., 2008; Rinne et al., 2013; Sahlholm, Marcellino, Nilsson, Fuxe, & Arhem, 2008) of agonists. In the case of the μ receptor, voltage‐induced changes in the morphine‐induced activation of μ receptors can be attributed to alterations of ligand efficacy. This conclusion was supported by three findings obtained from our study: first, the depolarization‐induced increase in morphine‐evoked, μ receptor‐mediated GRK2 and arrestin3 recruitment occurs even upon application of saturating concentrations of morphine (Figures 5a and 6a,c). Second, the concentration–response curve for morphine in the GRK2 recruitment assay for +30 mV showed a robust increase in the maximum effect compared to −90 mV while no significant change in the EC_50_ was observed (Figure 5b). Third, comparison of relaxation kinetics of the arrestin3–μ receptor interaction signal, with μ receptor–arrestin3 dissociation upon agonist withdrawal showed a twofold faster dissociation of the complex upon repolarization of V_M,_ as compared to acute agonist withdrawal (Figure S6C–E).

Based on our results that show a decrease of fentanyl‐induced GRK2 recruitment (Figure 5e) to μ receptors upon depolarization, even in the presence of saturating concentrations of agonist, we propose that voltage also decreases the efficacy of fentanyl to activate μ receptors. Interestingly, for arrestin recruitment, we could only observe decreased efficacy of fentanyl with sub‐saturating concentrations (Figures 6e and S6G), which suggests a different sensitivity of the two assays for depolarization‐induced changes in signalling. For DAMGO, the effect of voltage was reduced, but not abolished, upon application of saturating concentrations, suggesting effects on efficacy and affinity (Figures 5c and 6c). We predict that depolarization affects the conformation of the ligand binding pocket of the μ receptor and the resulting alterations in ligand binding give rise to a ligand‐specific modulation of μ receptor activation. It is interesting to note that voltage‐induced changes in ligand efficacy were not correlated with intrinsic efficacy of the compounds themselves. The order of intrinsic efficacy of the agonists used in this study is DAMGO > ME > fentanyl > morphine (McPherson et al., 2010), whereas the rank order of efficacy increase by depolarization is morphine > DAMGO > ME > fentanyl. The finding that voltage affects μ receptor activity in a ligand‐specific manner might be interesting not only for understanding the molecular mechanism responsible but also has therapeutic implications as voltage sensitivity might be an additional determinant of the clinical profile of opioid receptor agonists.

### 4.2 Morphine‐induced activation of μ receptors exhibits strong voltage‐dependent regulation

In the presence of morphine, voltage‐dependent changes were apparent across the physiological range of membrane potentials and were of large magnitude in each of the assays used. Voltage sensitivity of μ receptors was apparently quantitatively comparable in all signalling levels, independent of the method and assay system used: K_ir_3.X currents were measured by means of whole‐cell patch‐clamp recording both in HEK293T cells and in LC neurons from brain slices and GRK2 or arrestin3 recruitment, as well as G‐protein activation was measured by means of single cell FRET assays under conditions of voltage‐clamp. Detectable effects of voltage on μ receptor activity were only seen in the presence of agonist (Figure S4B, S5A and S6B).

Even in native tissue, which required no overexpression of interacting proteins or fluorescent labelling, we could observe a significant effect of depolarization on morphine‐induced μ receptor signalling, despite experimental limitations of the LC slices (North & Williams, 1985) which allowed only a small voltage range between −80 and −40 mV. Consequently, the voltage sensitivity must be an intrinsic property of the μ receptor itself. The response/depolarization relationship obtained from morphine data in the arrestin3 recruitment assay followed a Boltzmann function (Figure 6b). The obtained slope of the fit can be used to calculate the membrane potential of half‐maximum depolarization‐induced response (V_0.5_ value) which was within the physiological range of membrane potential. The calculated net charge movement (*z*‐factor: 0.66) obtained from the slope of the Boltzmann function of arrestin3 recruitment was similar to previously published values from muscarinic receptors (Ben‐Chaim et al., 2006; Navarro‐Polanco et al., 2011) or adrenoceptors (Birk et al., 2015; Rinne et al., 2013). This charge movement is caused by the reorientation of one or more charges across the receptor molecule, which thereby could lead to both changes in affinity (Figures 5c and 6c) and efficacy (Figures 5a,b, 6a,c and S6C,D), as we observed both for the μ receptors. Moreover, the magnitude of the voltage‐induced changes of the morphine response was large compared to other GPCRs (about 4.9‐fold increase upon depolarization of the μ receptors to +60 mV in arrestin3 recruitment vs. changes of up to twofold upon repolarization [as depolarization there led to a deactivation] from the same voltage step in ß_1_‐ or α_2A_‐adrenoceptors; Birk et al., 2015; Rinne et al., 2013).

### 4.3 Voltage sensitivity of μ receptors is also reflected in downstream signalling

One of the physiological effects of μ receptor‐induced G_i/o_ activation is the opening of K_ir_3.X channels, which leads to hyperpolarization, decreased neuronal excitation and reduced propagation of action potentials (Lüscher & Slesinger, 2010; Stein, 2016). The finding of voltage insensitivity of ME allowed for comparison of voltage‐induced alterations in responses to morphine relative to those induced by ME in LC neurons (Figure S3B). Our results show that in both HEK293T cells and LC neurons, even a moderate depolarization enhanced the amplitude of morphine‐evoked K_ir_3.X currents relative to the response evoked by ME or DAMGO. In HEK293T cells, we observed a robust 2.5‐fold increase in the proportional morphine‐induced K_ir_3.X activation upon depolarization from −50 to 0 mV (Figure 2a,b), which compares well with our results using FRET‐based assays for G‐protein activation, GRK2 or arrestin3 binding. Detecting voltage‐dependent modulation of GPCRs in neurons is more challenging than in HEK cells, and we were thus limited to examining membrane potentials between −80 and −40 mV (North & Williams, 1985) Also, given the relatively shallow nature of the response/depolarization relationship (Figure 6b), we could only expect relatively small voltage‐dependent changes in morphine‐mediated μ receptor activity in LC neurons. However, we were able to demonstrate a proportional and significant enhancement of morphine‐mediated K_ir_3.X currents in LC neurons upon depolarization from −80 to −40 mV, compared to those evoked by the voltage‐insensitive agonist ME (Figures 3b,c and S3D). Furthermore, in two control experiments, we could rule out time‐ or protocol‐dependent effects on this finding (Figure S3A–D). The finding of the increased potency of morphine in excited neurons is important, as it helps develop our understanding of the implications of voltage sensitivity in physiological tissue and might contribute to tissue‐specific differences of morphine‐mediated signalling (Haberstock‐Debic, Kim, Yu, & von Zastrow, 2005). Furthermore, our findings that peptide μ receptor agonists exhibited minimal voltage dependence in LC neurons suggest minimal effects of voltage on physiological μ receptor function, when activated via endogenous agonists, compared with morphine. We propose that both analgesic potency and side effects of these drugs will partly depend on their voltage sensitivity, as fentanyl should be considerably more potent in μ receptor‐expressing neurons with low electrical activity (hyperpolarized), compared with morphine which should show increased potency in excited (depolarized) tissue. It is posible that some of the observed differences in the clinical profile of these analgesics, such as increased mortality (via respiratory depression) (Ferrante, 1993) but reduced nausea, with fentanyl (Khaled, Tafish, & Zourob, 2018) at equal analgesic doses, could be attributed to their differences in their voltage‐dependence.

Taken together, the pronounced voltage sensitivity of the morphine‐activated μ receptor that we have observed in transfected cells, as well as in native neurons, demonstrates the importance of voltage sensitivity of GPCRs in living systems and suggests ligand‐specific voltage sensitivity as a novel opportunity for drug development.

## CONFLICT OF INTEREST

The authors declare no conflicts of interest.

## AUTHOR CONTRIBUTIONS

J.G.R. and S.B.K. designed, performed and analysed the FRET and electrophysiological measurements in HEK293T cells, S.K. performed the experiments in brain slices and J.G.R and S.K. analysed data from brain slices. M.B. designed the study and experiments. J.G.R. wrote the manuscript, and M.B., S.B.K. and C.B. reviewed and edited the manuscript.

## DECLARATION OF TRANSPARENCY AND SCIENTIFIC RIGOUR

This Declaration acknowledges that this paper adheres to the principles for transparent reporting and scientific rigour of preclinical research as stated in the *BJP* guidelines for Design & Analysis, and Animal Experimentation, and as recommended by funding agencies, publishers and other organizations engaged with supporting research.

## Supporting information

Figure S1: Membrane potential regulates MOP‐induced outward GIRK currents
Figure S2: The observed voltage sensitivity occurs also in physiological K^+^ concentrations
Figure S3: Comparison of voltage induced current responses in LC neurons
Figure S4: Bleach correction of FRET recordings, depolarization in the absence of agonist, Reproduction of the protocol from GIRK recordings in FRET recordings from G‐protein activation, comparison of voltage induced changes in Go‐ and Gi‐protein activation assay
Figure S5: Depolarization in the absence of agonist, single emission traces and Bystander FRET experiments in a GRK2‐recruitment assay
Figure S6: Single emission traces and depolarization in the absence of agonists, kinetics of voltage‐induced changes, characterization of voltage induced effects on DAMGO‐ or fentanyl‐mediated Arrestin3 recruitment at equieffective concentrations to morphine
