# Supplementary material for: Voltage modulates the effect of μ‐receptor activation in a ligand‐dependent manner
Source: Br J Pharmacol. 2020 May 19;177(15):3489–504. doi: 10.1111/bph.15070 (PMC7348086; doi:10.1111/bph.15070)
Supplement: Supplementary file 1 — Figure S1: Membrane potential regulates MOP‐induced outward GIRK currents Figure S2: The observed voltage sensitivity occurs also in physiological K+ concentrations Figure S3: Comparison of voltage induced current responses in LC neurons Figure S4: Bleach correction of FRET recordings, depolarization in the absence of agonist, Reproduction of the protocol from GIRK recordings in FRET recordings from G‐protein activation, comparison of voltage induced changes in Go‐ and Gi‐protein activation assay Figure S5: Depolarization in the absence of agonist, single emission traces and Bystander FRET experiments in a GRK2‐recruitment assay Figure S6: Single emission traces and depolarization in the absence of agonists, kinetics of voltage‐induced changes, characterization of voltage induced effects on DAMGO‐ or fentanyl‐mediated Arrestin3 recruitment at equieffective concentrations to morphine [file BPH-177-3489-s001.pdf]

Supplementary Material:

Opioid analgesics activate μ-opioid receptors in a voltage dependent manner

Julia G. Ruland[1], Sina B. Kirchhofer[1], Sebastian Klindert[1,2], Chris P. Bailey[2] & Moritz Bünemann[1*]

[1] Department of Pharmacology and Clinical Pharmacy, Philipps-University D-35043 Marburg, Germany.

[2] Department of Pharmacy and Pharmacology, University of Bath, Bath BA2 7AY, United Kingdom.

*To whom correspondence should be addressed: Email: moritz.buenemann@staff.uni-marburg.de.

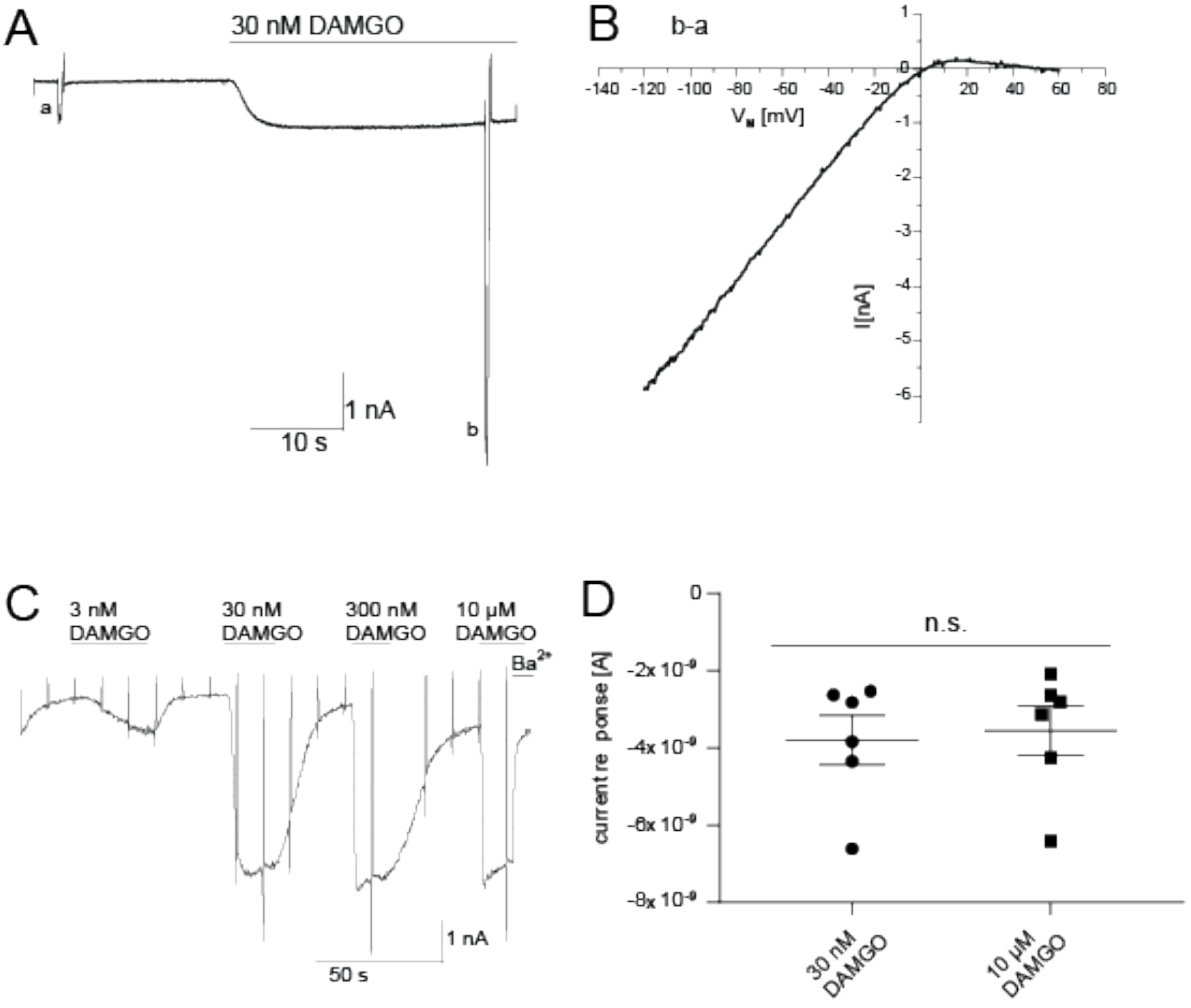

Figure S1: Membrane potential regulates MOP-induced outward GIRK currents. ***A,*** Magnification of the excerpt, which was used for calculation of the background substracted I/V curve (shown in B). Voltage ramps which were used for calculation are marked as a and b. ***B***, Background subtracted I/V-curve (b-a, obtained from the voltage ramps a and b), as indicated in *fig. S1A* show strong inward rectification**.** ***C,*** Representative recording (n = 6) of GIRK current responses to increasing concentrations of DAMGO. ***D,*** Statistical comparison of GIRK current responses to 30 nM and 10 µM DAMGO. As responses were compared withon the same cell statistics were performed using a paired t-test (n.s.: $p > 0.05$, n = 6).

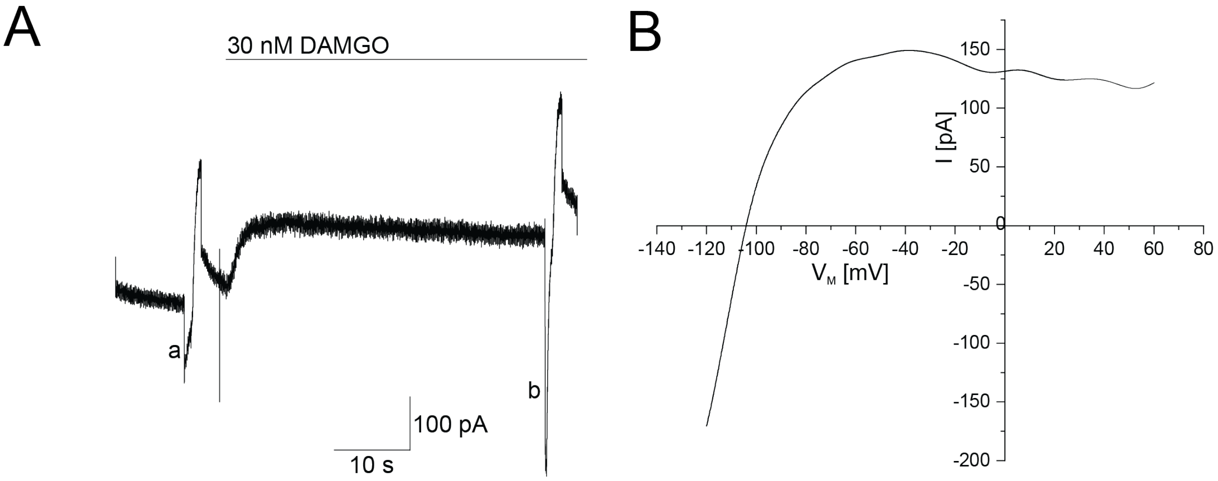

Fig. S2: The observed voltage sensitivity occurs also in physiological K^+ concentrations. ***A*****,** Magnification of the excerpt, which was used for calculation of the background substracted I/V-curve (shown in ***B***). Voltage ramps which were used for calculation are marked as a and b. ***B***, Background subtracted I/V-curve (b-a, obtained from the voltage ramps a and b), as indicated in *fig. S2A*.

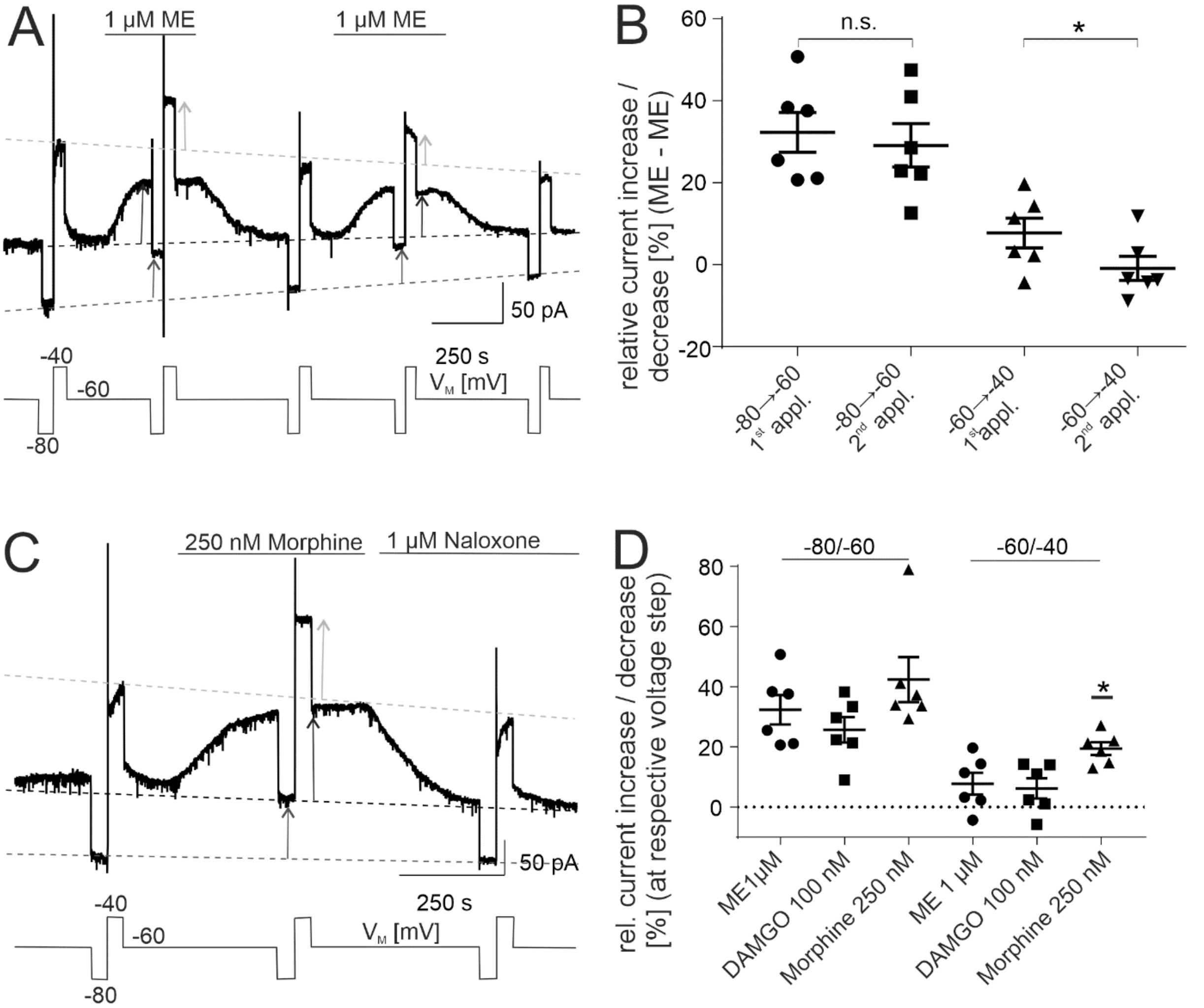

Figure S3: Comparison of voltage induced current responses in LC neurons. ***A,*** Representative recording of outward K^+ currents in LC neurons. GIRK currents were evoked by non-saturating concentration of Met-enkephalin (ME; 1 µM). During measurements, cells were kept at -60 mV and briefly clamped to -80 mV and -40 mV in turn (voltage protocol is depicted at the bottom, note that depolarization steps shift both basal and active currents due to the current-voltage relationship of the channel). Baseline current responses at each membrane potential (dashed lines) were determined by voltage-steps in control aCSF. The magnitude of respective agonist induced current responses are depicted as arrows (dark grey: -80 mV; black: -60 mV, light grey: -40 mV). ***B,*** Statistical analysis in voltage induced current change for 2 subsequent applications of Met-enkephalin (ME) shows that second applications of ME cause significantly smaller current responses (**p:* < 0.05; n = 6; paired t-test). ***C,*** Representative recording of morphine (250 nM) induced responses upon single ligand application in LC neurons. During measurements, cells were kept at -60 mV and briefly clamped to -80 mV and -40 mV in turn (voltage protocol is depicted at the bottom, note that depolarization steps shift both basal and active currents due to the current-voltage relationship of the channel). Baseline current responses at each membrane potential (dashed lines) were determined by voltage-steps in control aCSF. To accelerate wash out of morphine, naloxone (1 µM) was applied afterwards. The magnitude of respective agonist induced current response is depicted as arrows (dark grey: -80 mV; black: -60 mV, light grey: -40 mV). ***D*** Voltage-induced current changes during single agonist applications of Met-enkephalin (ME), DAMGO or morphine (as shown in fig. S3C, *: $p < 0.05$, ordinary one-way ANOVA with Tukey's multiple comparison test; n = 6 respectively) show that relative increase of currents in the -60 to -40 depolarization step are significantly higher during morphine application. Note, that due to the current voltage relationship a general increase in ligand-evoked currents can be seen upon depolarization, from which the increase seen upon morphine application differs statistically significantly.

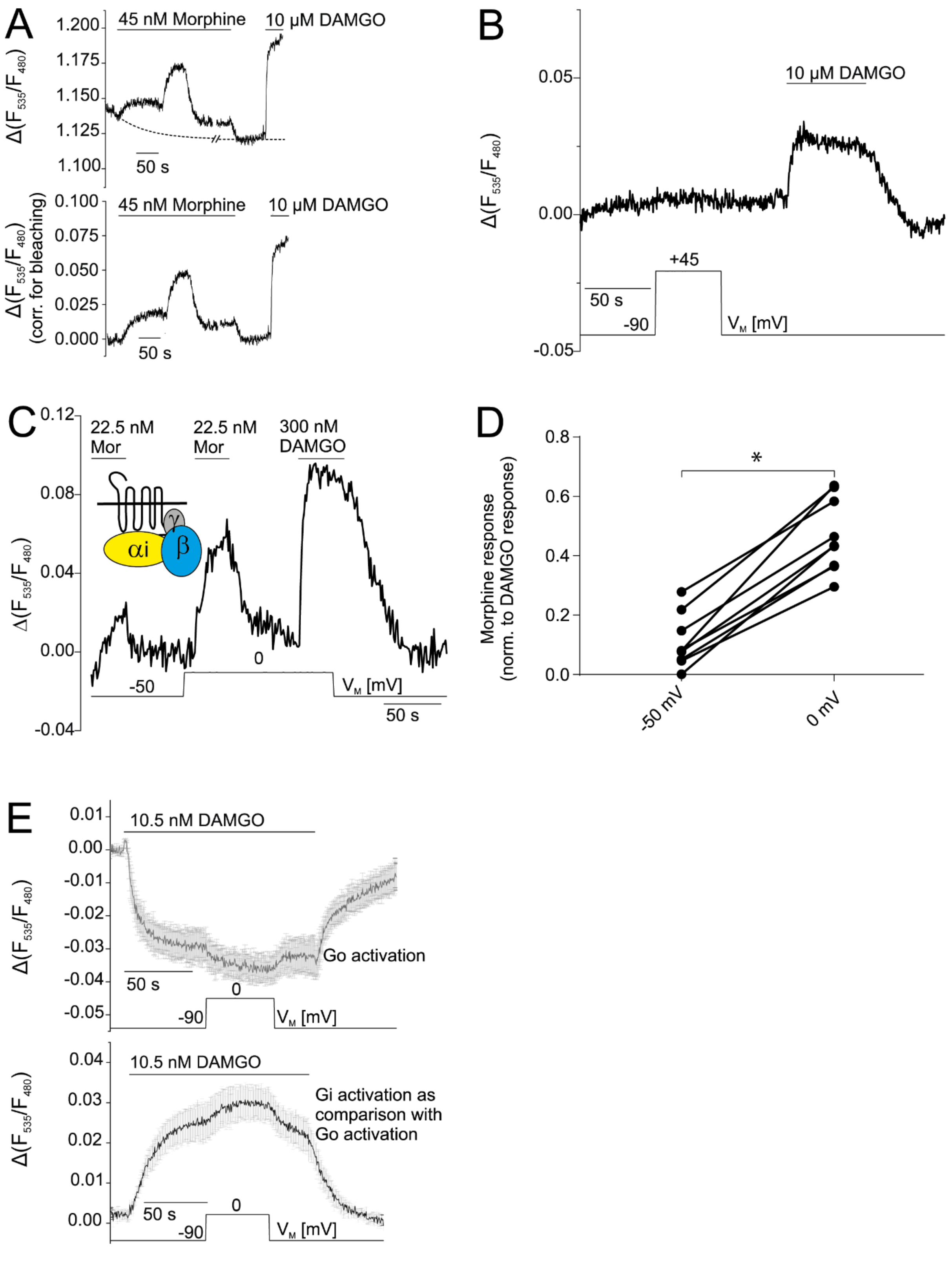

Figure S4: Bleach correction of FRET recordings, depolarization in the absence of agonist, Reproduction of the protocol from GIRK recordings in FRET recordings from G-protein activation, comparison of voltage induced changes in Go- and Gi-protein activation assay, ***A,*** Representative FRET trace of a cell that was transiently transfected with unlabeled MOP, Gαi-YFP, Gß$_1$-mTur2 and Gγ without correction for photobleaching (above) and after subtraction with a mono-exponential function (below). ***B,*** Representative cell (out of n = 5) that was transfected with unlabeled

MOP, Gαi-YFP, Gß$_1$-mTur2 and Gγ. A depolarization step from a holding potential of -90 mV to +45 mV was performed upon buffer application and for control reasons, a saturating concentration of agonist (McPherson et al., 2010) was applied subsequently. ***C,*** A representative FRET recording (out of n=9) from cells that were transfected with MOP-wt, Gα$_i$-YFP, Gß$_1$-mTur2, Gγ$_2$-wt is illustrated. Morphine- or DAMGO-mediated G-protein activation is shown by an increase in FRET (Bünemann et al., 2003) measured at -50 mV and 0 mV. Traces were corrected for photobleaching as indicated in *fig. S3A*. ***D,*** In analogy to GIRK current measurements (as conducted in Fig. 2), the same protocol was used to determine G-protein activation evoked by an non-saturating morphine concentration (22.5 nM) was normalized to the maximum response (300 nM DAMGO) at respective membrane potentials (n=9) and the relative voltage-dependent effect induced by morphine was compared at -50 mV and 0 mV (*$p <$ 0.05 between responses at -50mV and 0mV; paired t-test, as responses were compared within the same cell). ***E,*** Averaged (mean ± S.E.M., n = 9 for Gi, n = 3 for Go) preliminary traces of agonist/voltage evoked alterations in FRET are shown and compared for cells expressing the Go –FRET sensor (upper panel) or the Gi-protein-FRET sensor (Bünemann et al., 2003; Frank et al., 2005). ***F,***

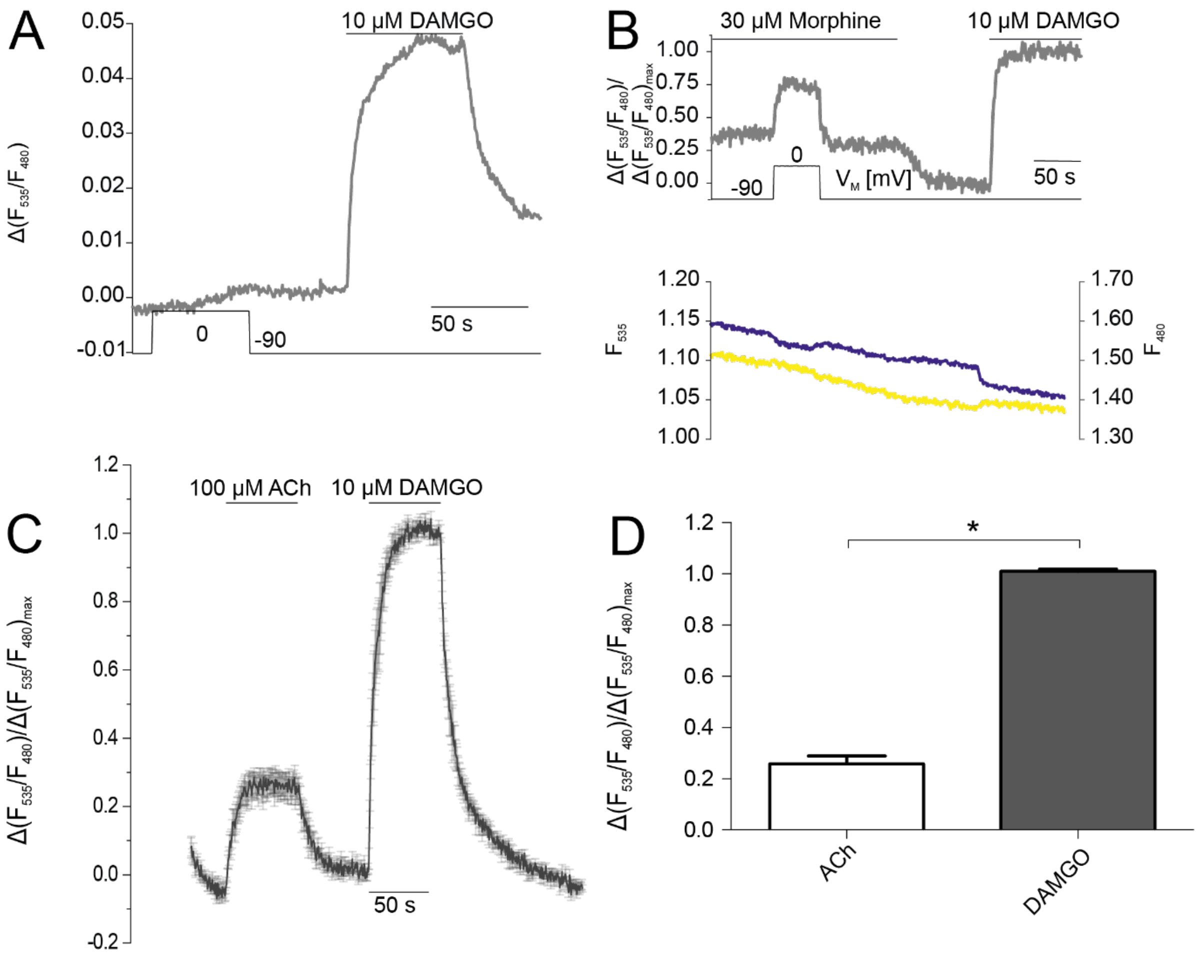

Figure S5: Depolarization in the absence of agonist, single emission traces and Bystander FRET experiments in a GRK2-recruitment assay. ***A***, Representative FRET (out of n = 6) recording from cells that were transfected with sYFP2-labelled MOP and mTur2-labelled GRK2, as shown previously. Depolarization in the absence of agonist has no effect on MOP – GRK2 interactions. ***B***, Representative FRET recording plotted as relative agonist evoked alterations in the sYFP2/mTur2 emission ratio and corresponding recordings of CFP (F_{480}) and YFP (F_{535}) emission. ***C***, Bystander FRET between MOP-sYFP2 and GRK2-mTur2 evoked by 100 µM ACh at non-labelled muscarinic M_3R leads to 26 % ± 3 % (mean ± S.E.M.) non-specific FRET (compared to a maximum DAMGO response (n = 9), ***D***, Statistical analysis of FRET amplitudes following non-specific, ACh-evoked response and DAMGO-induced response. To compare responses within the same cell, a paired t-test was used (*: $p < 0.05$).

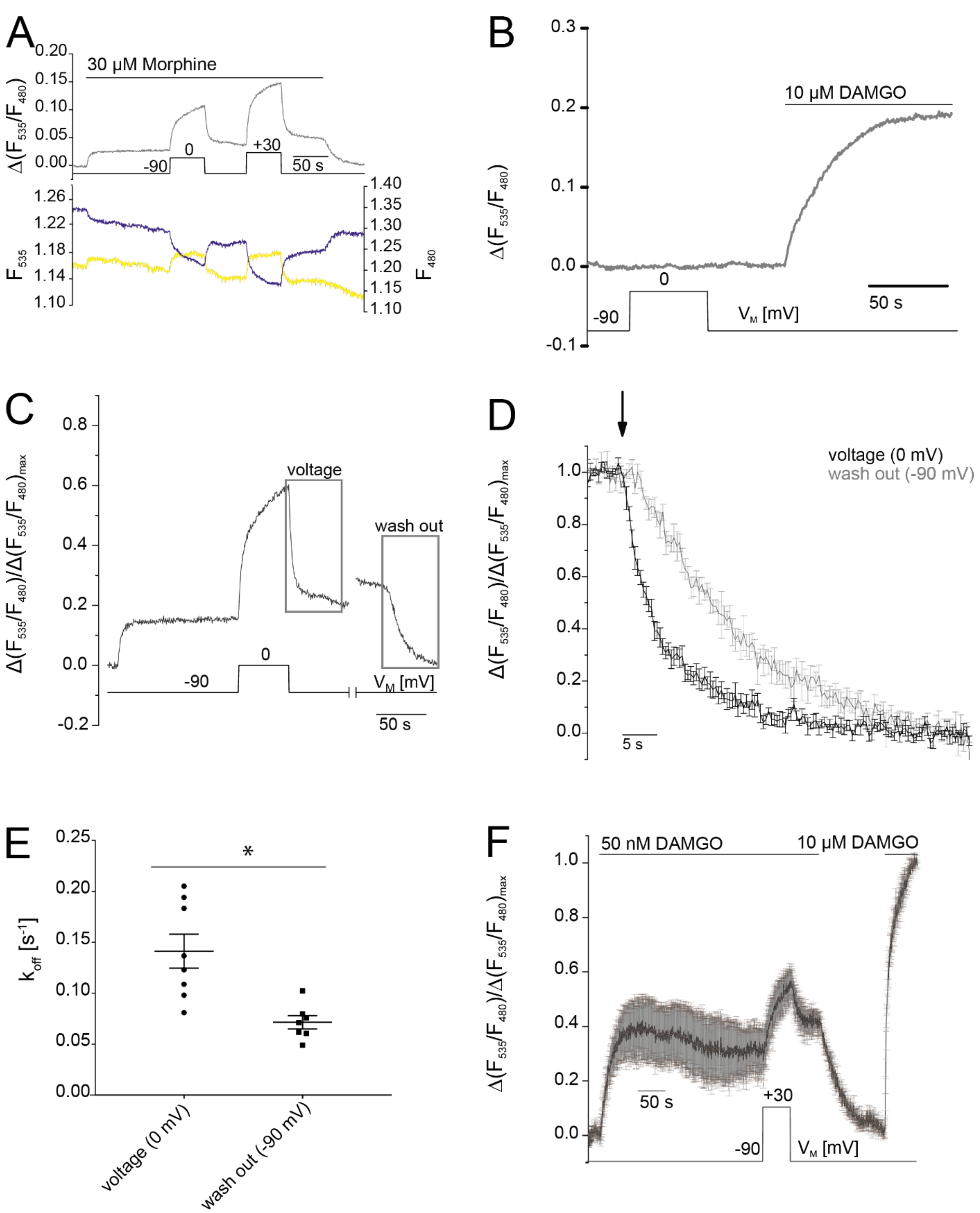
A
30 µM Morphine
Δ(F535/F480)
0.20
0.15
0.10
0.05
0.00
-90
0
+30
50 s
F535
F480
1.26
1.22
1.18
1.14
1.10
1.40
1.35
1.30
1.25
1.20
1.15
1.10
B
10 µM DAMGO
Δ(F535/F480)
0.3
0.2
0.1
0.0
-0.1
-90
0
VM [mV]
50 s
C
Δ(F535/F480)/Δ(F535/F480)max
voltage
wash out
0.8
0.6
0.4
0.2
0.0
-0.2
-90
0
VM [mV]
50 s
D
voltage (0 mV)
wash out (-90 mV)
Δ(F535/F480)/Δ(F535/F480)max
1.0
0.8
0.6
0.4
0.2
0.0
5 s
E
koff [s-1]
0.25
0.20
0.15
0.10
0.05
0.00
*
voltage (0 mV)
wash out (-90 mV)
F
50 nM DAMGO
10 µM DAMGO
Δ(F535/F480)/Δ(F535/F480)max
1.0
0.8
0.6
0.4
0.2
0.0
50 s
+30
-90
VM [mV]

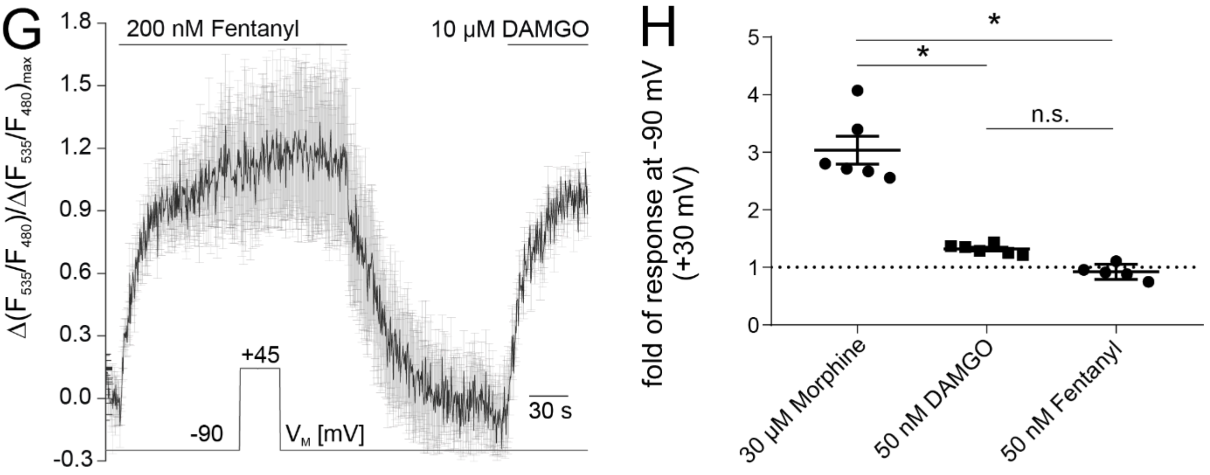

Figure S6: Single emission traces and depolarization in the absence of agonists, kinetics of voltage-induced changes, characterization of voltage induced effects on DAMGO- or fentanyl-mediated Arrestin3 recruitment at equieffective concentrations to morphine. ***A***, Representative FRET recording plotted as relative agonist evoked alterations in the sYFP2/mTur2 emission ratio and corresponding recordings of CFP (F_{480}) and YFP (F_{535}) emission. ***B***, Voltage does not influence MOP – Arrestin3 interactions in the absence of agonist (representative FRET trace, n = 7). ***C***, Exemplary trace (of n = 7) indicates which excerpts were used for determination of kinetic analysis (see fig. S6D, E) of depolarization-induced or wash-out induced termination of MOP-Arrestin interaction. ***D***, Average (mean ± S.E.M.) recordings showing termination of the MOP-Arrestin3-interaction upon wash-out (light grey trace, n = 7) of 30 µM morphine at holding potential (-90 mV) or following repolarization (black trace, n = 8) from 0 mV to holding potential (-90 mV) in continued application of 30 µM morphine. The arrow indicates the time point of agonist withdrawal or repolarization respectively. ***E***, Comparison and statistical analysis of k_{off}-values (for data see *C*). Statistical significance was determined by a paired t-test as responses were compared within same cells (*: $p < 0.05$; n = 7). ***F,*** Average (mean ± S.E.M.; n = 6) of MOP-Arrestin interaction upon application of 50 nM DAMGO (sub-saturating concentration). Cells were depolarized from -90 mV to +30 mV. ***G***; Average (mean ± S.E.M.; n = 4) of MOP-Arrestin interaction upon application of 200 nM fentanyl, saturating concentration. Cells were depolarized from -90 mV to +45 mV.***H,*** Statistical analysis of changes in MOP-Arrestin3 interaction upon depolarization from -90 mV to +30 mV under application of a saturating concentration morphine or a non-saturating concentration of DAMGO or fentanyl (Data shown in Fig. 6E). To confirm statistically significant differences in voltage dependent response for morphine, DAMGO and fentanyl an ordinary one-way ANOVA with Tukey's multiple comparison test was used (*: $p < 0.05$; n.s.: $p > 0.05$; n = 5 - 6).
